# Differential Roles of Angiotensin A and Alamandine in Parkinson's Disease: A Therapeutic Perspective on Nonclassical RAS Pathways

**DOI:** 10.1002/brb3.70721

**Published:** 2025-08-12

**Authors:** Ghadah H. Alshehri, Hayder M. Al‐kuraishy, Ali K. Albuhadily, Ali I. Al‐Gareeb, Amany S. Aboutaleb, Athanasios Alexiou, Marios Papadakis, Gaber El‐Saber Batiha

**Affiliations:** ^1^ Department of Pharmacy Practice, College of Pharmacy Princess Nourah bint Abdulrahman University Riyadh Saudi Arabia; ^2^ Department of Clinical Pharmacology and Medicine, College of Medicine Mustansiriyah University Baghdad Iraq; ^3^ Jabir Ibn Hayyan Medical University Qu./Najaf Iraq; ^4^ Department of Pharmacology and Toxicology, Faculty of Pharmacy (Girls) Al‐Azhar University Cairo Egypt; ^5^ University Centre for Research & Development Chandigarh University Mohali Punjab India; ^6^ Department of Research & Development Funogen Athens Attiki Greece; ^7^ University Hospital Witten‐Herdecke, Heusnerstrasse 40 University of Witten‐Herdecke Wuppertal Germany; ^8^ Department of Pharmacology and Therapeutics, Faculty of Veterinary Medicine Damanhour University Damanhour AlBeheira Egypt

**Keywords:** alamandine, angiotensin, angiotensin‐converting enzyme 2, Parkinson's disease, renin–angiotensin system

## Abstract

Background: Parkinson's disease (PD) is a neurodegenerative disease characterized by progressive neurodegeneration of dopaminergic neurons (DNs) in the substantia nigra (SN). PD neuropathology is mainly related to inflammation, mitochondrial dysfunction, oxidative stress, and endoplasmic reticulum (ER) stress. The underlying causes for the progression of PD are linked to the uncontrolled activation of different signaling pathways, such as the renin–angiotensin system (RAS), which is highly expressed in the nigrostriatal pathway. RAS has two main pathways: the classical pathway, which includes angiotensin I (AngI), AngII, angiotensin‐converting enzyme (ACE), Ang type 1 receptor (AT1R), and Ang type 2 receptor (AT2R), that has a neuro‐detrimental effect on PD neuropathology, and the nonclassical pathway, which includes angiotensin‐converting enzyme 2 (ACE2)/Angiotensin 1–7 (Ang1–7), that has a neuroprotective effect against different neurological disorders, including PD. The nonclassical pathway is activated to overcome the harmful impact of the classical pathway on the brain, thereby converting AngII to angiotensin A (Ang‐A) via mononuclear leukocyte‐derived aspartate decarboxylase (MILDAD). Ang‐A is further converted to the neuroprotective alamandine, which acts on the mass‐related G‐protein coupled receptor member D (MrgD). In PD, overactivation of the classical pathway is associated with neurodegeneration of the DNs in the SN. However, the nonclassical pathway, mainly Ang1–7/alamandine, is deregulated in PD. The objective of the review: This review aims to explore and discuss the potential role of the nonclassical RAS pathway in the pathogenesis and progression of PD and its implications for future therapeutic strategies.

## Graphical abstract

The role of the Classical and Non‐Classical RAS pathway in Parkinson's disease: Pathogenesis and Therapeutic strategies. PD, Parkinson's disease; DNs, Dopaminergic neurons; SN, Substantia Nigra; α‐syn, alpha‐synuclein; RAS, renin‐angiotensin system; Ang II, Angiotensin II; AT1R, Angiotensin II type 1 Receptor; ACE2, angiotensin‐converting enzyme 2; Ang 1‐7, Angiotensin 1‐7; Ang A, Angiotensin A.

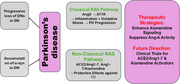

## 1 Introduction

**FIGURE 1:**
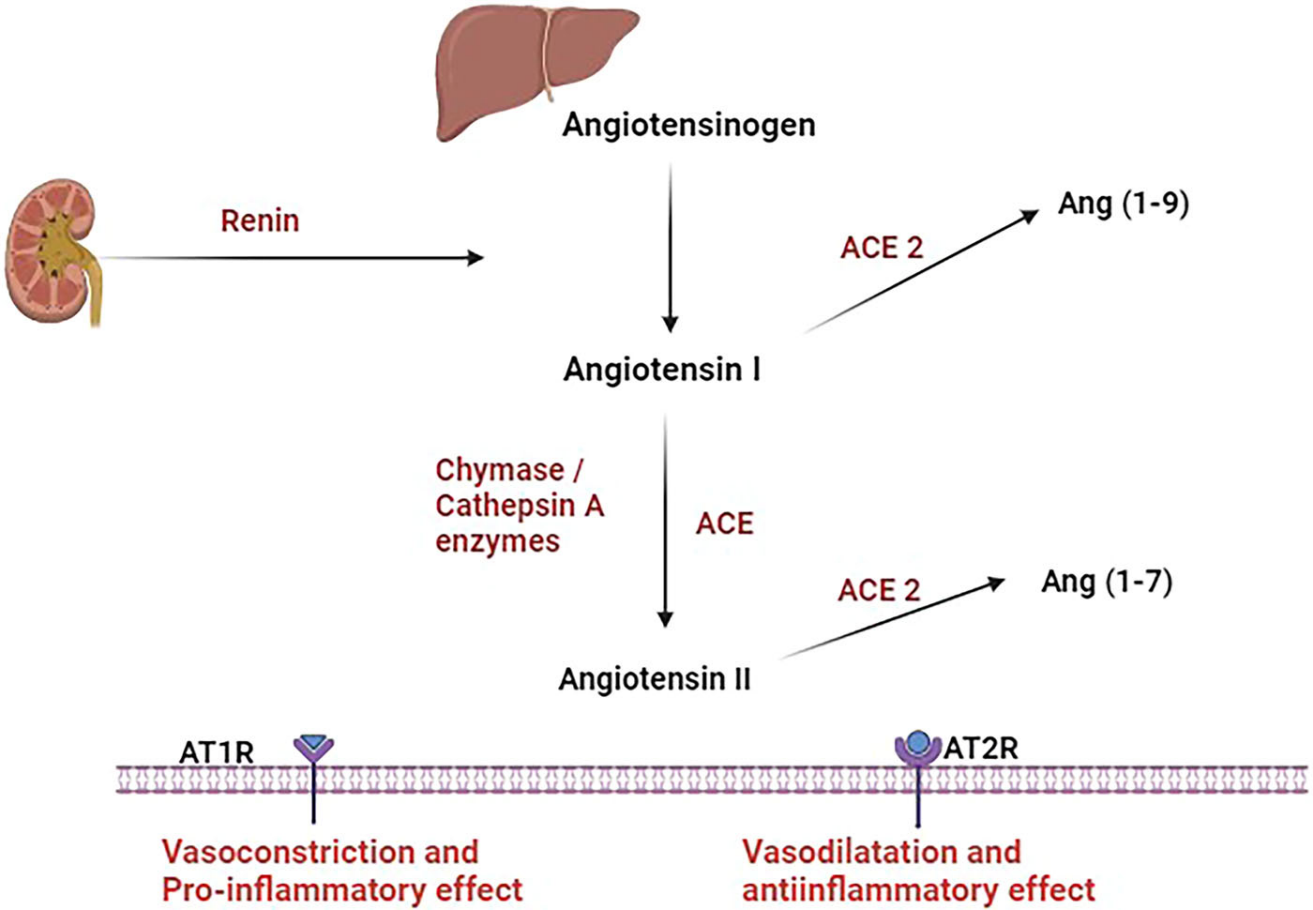
Pathway of RAS: Hepatic angiotensinogen is converted by the effect of renal renin to angiotensin I (AngI), which is converted to AngII by the impact of angiotensin‐converting enzyme (ACE), chymase, and cathepsin A. AngII acts on angiotensin type 1 receptor (AT1R) and angiotensin type 2 receptor (AT2R), leading to vasoconstriction, pro‐inflammatory effect and vasodilation and anti‐inflammatory effect respectively. In addition, AngI and AngII are metabolized via ACE2 to the Ang (1–7) and Ang (1‐9), respectively.

The renin–angiotensin system (RAS) regulates body water, minerals, and blood pressure (Ali et al. 2024). The liver produces angiotensinogen, the primary precursor for angiotensin I (AngI) that is converted by renin from the kidney to AngI (Al‐Kuraishy, Hussien, et al. 2020). Angiotensin‐converting enzyme (ACE) then converts AngI to AngII, which acts on AngII receptors known as AT1R and AT2R. Chymase and cathepsin enzymes also convert AngI to AngII. The AT1R receptor triggers constriction of blood vessels and promotes pro‐inflammatory effects, while the AT2R receptor mediates the vasodilatory and anti‐inflammatory actions of AngII. ACE2 also converts AngI to change into Ang (1–9) and AngII to change into Ang (1–7), which activates the MAS receptor and causes an anti‐inflammatory response (Al‐Qahtani et al. 2024) (Figure 1).

**FIGURE 2:**
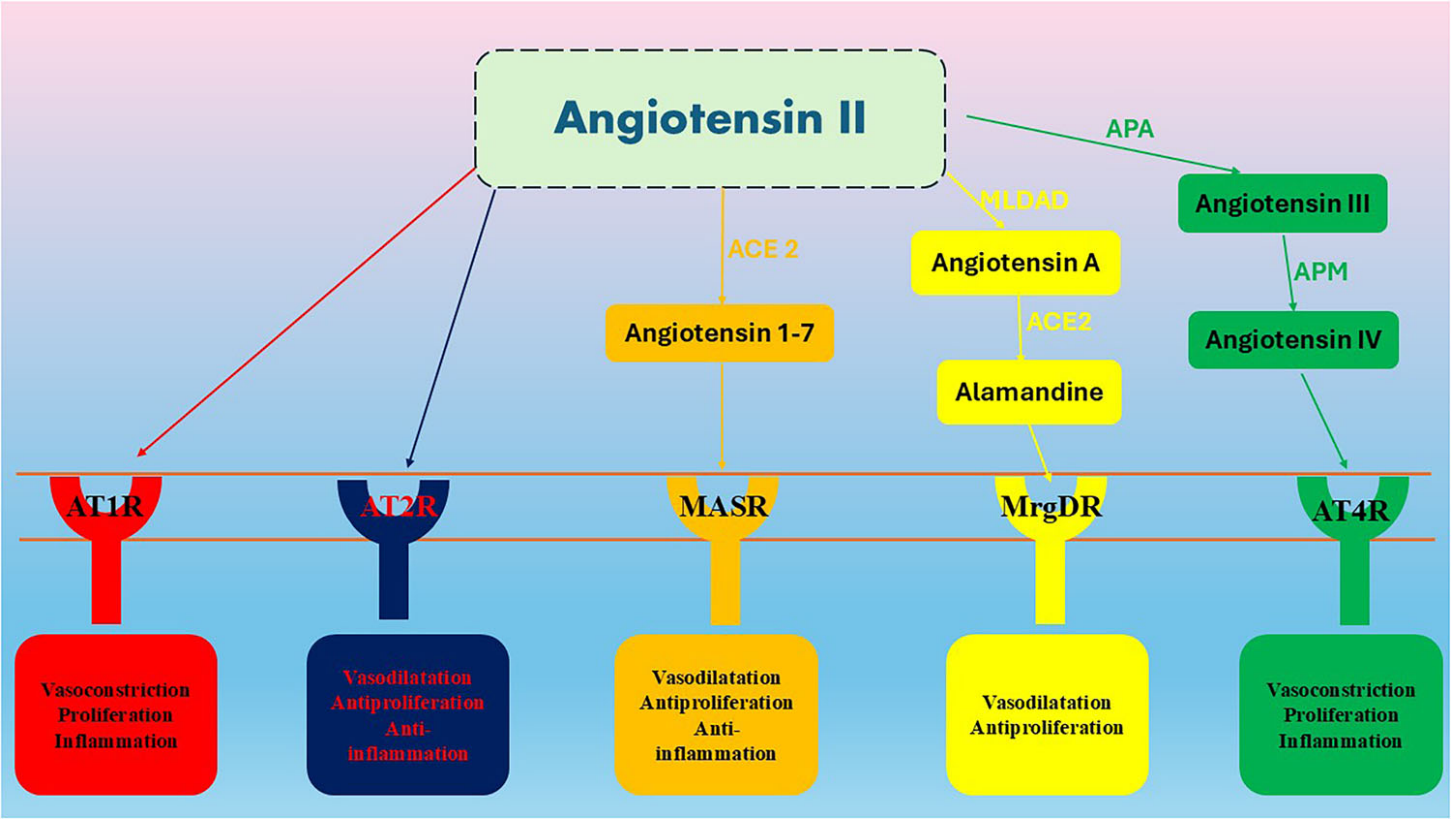
Pathway of angiotensin II: AngII acts on angiotensin type 1 receptor (AT1R) and angiotensin type 2 receptor (AT2R), leading to vasoconstriction, pro‐inflammatory effect, and vasodilation and anti‐inflammatory effect, respectively. Ang (1–7) activates the MAS receptor (MASR), resulting in vasodilation, antiproliferative, and anti‐inflammatory effects. AngII can be converted to Ang‐A by mononuclear leukocyte‐derived aspartate decarboxylase (MLDAD). ACE2 then further converts Ang‐A to alamandine. As well, alamandine may be derived directly from Ang1–7. Agn‐A acts on AT1R, while alamandine activates mass‐related G‐protein‐coupled receptor member D (MrgD), resulting in vasodilation and antiproliferative effects. In addition, AngIII, which is derived from AngII by the effect of aminopeptidase (APA), is metabolized to AngIV by the effect of aminopeptidase M (APM). AngIV, by activating AT4R, leads to vasoconstriction, proliferation, and inflammation.

The RAS can produce systemic and local effects as well as paracrine effects (Al‐Kuraishy et al. 2019). In various tissues, the systemic and local RAS work together. The increased AngII conversion and activation of AT1R stimulate the onset of oxidative stress, free radicals, and inflammation by initiating NADPH oxidase, which leads to mitochondrial dysfunction (Al‐Kuraishy, Al‐Gareeb, et al. 2020). The expression of NADPH oxidase is significantly increased during aging and in age‐related conditions, such as diabetes and hypertension (Rungratanawanich et al. 2021). However, the activation of AT2R by AngII suppresses inflammation and oxidative stress (Mei et al. 2022). There are two main pathways of RAS (Table 1): the classical pathway involves AngII/AT1R, leading to vasoconstriction and pro‐inflammatory changes, and the nonclassical pathway involves AngII/AT2R and ACE2/Ang1–7/MASR, leading to vasodilatory and anti‐inflammatory effects (Mei et al. 2022). Furthermore, AngII can be converted to Ang‐A by mononuclear leukocyte‐derived aspartate decarboxylase (MLDAD). ACE2 converts Ang‐A to alamandine, derived directly from Ang1–7 (Hrenak et al. 2016; Al‐Kuraishy, Al‐Gareeb, Alsayegh, et al. 2023; Alorabi et al. 2022; Batiha et al. 2021; Al‐Kuraishy, Al‐Gareeb, et al. 2022; Al‐Kuraishy and Al‐Gareeb, 2016; Rasheed et al. 2019). Agn‐A acts on AT1R, while alamandine activates mass‐related G‐protein‐coupled receptor member D (MrgD) (Hrenak et al. 2016). In addition, AngIII, derived from AngII by the effect of aminopeptidase (APA), is metabolized to AngIV by the effect of aminopeptidase M (APM). AngIV activates AT4R, leading to vasoconstriction, proliferation, and inflammation (Hrenak et al. 2016) (Figure 2).

**TABLE 1:** Classical versus nonclassical RAS pathways.

Characteristics	Classical pathway	Non‐classical pathway
Components Pathways Effects Neuroprotection Role in PD	AngI, ACE, AT1R, and AT2R (Mei et al. 2022). AngII/AT1R, AngII/AT2R (Sansoè et al. 2020). Pro‐inflammatory and oxidative effects (Mei et al. 2022). No Exaggerated (Al‐Kuraishy et al. 2018; Wright and Harding, 2019).	ACE2, Ang1–7, Ang1‐9, Agn‐A, alamandine, AngIII, and AngIV (Hrenak et al. 2016; Al‐Kuraishy, Al‐Gareeb, Alsayegh, et al. 2023; Alorabi et al. 2022; Batiha et al. 2021; Al‐Kuraishy et al. 2022; Al‐Kuraishy and Al‐Gareeb, 2016; Rasheed et al. 2019). ACE2/Ang1–7/MASR, Ang1–7/alamandine, Ang‐A/ACE2/Alamandine/MrgD (Hrenak et al. 2016). Anti‐inflammatory and antioxidant effects (Hrenak et al. 2016). Yes Reduced (Sunanda et al. 2021; Jo et al. 2022).

## 2 Brain RAS

**FIGURE 3:**
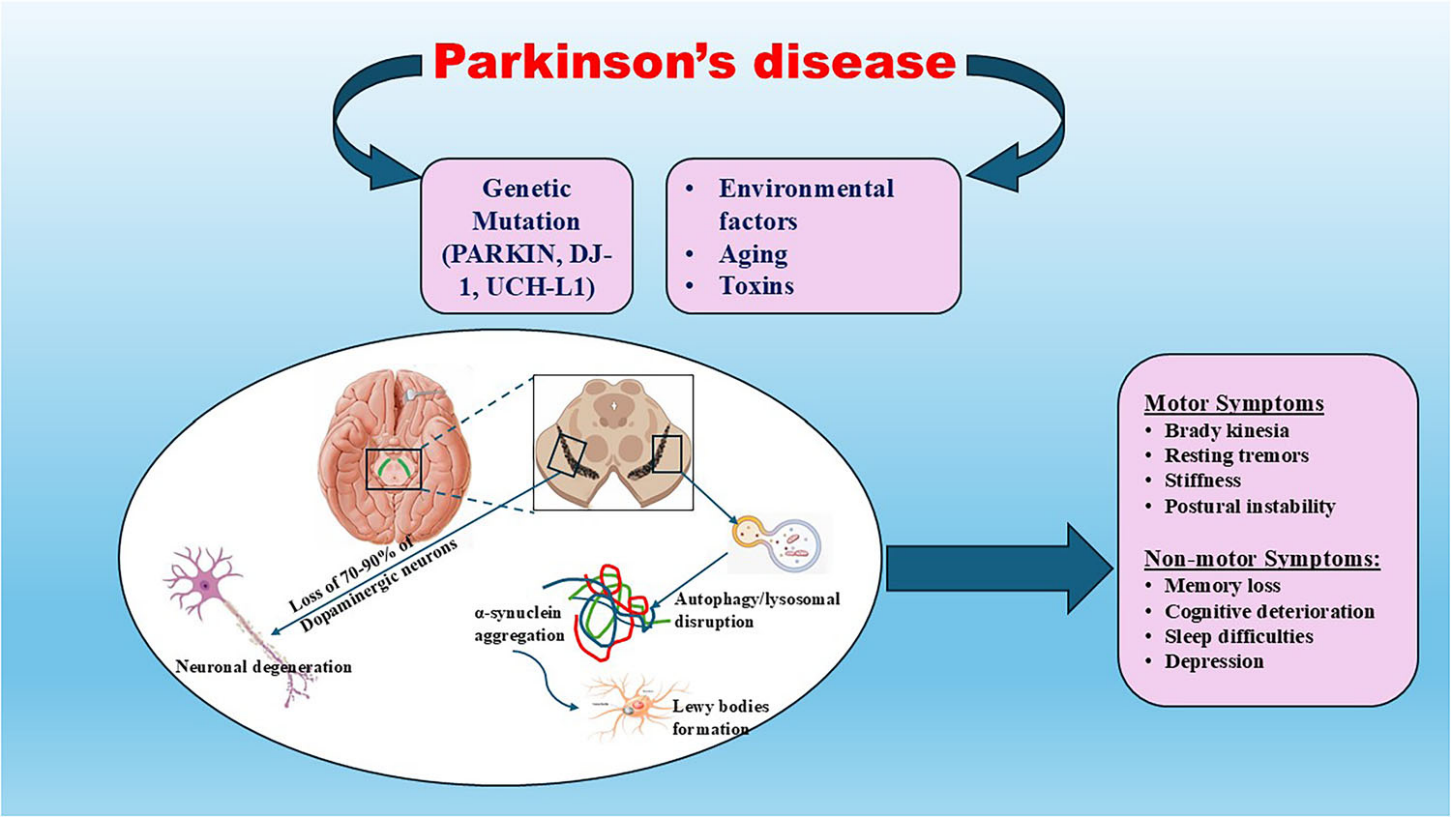
Pathway of brain RAS: Brain angiotensinogen is converted by the effect of renal renin to angiotensin I (AngI), which is converted to AngII by the effects of angiotensin‐converting enzyme (ACE). AngII acts on the angiotensin type 1 receptor (AT1R) and angiotensin type 2 receptor (AT2R), leading to vasoconstriction, a pro‐inflammatory effect, and vasodilation and an anti‐inflammatory effect, respectively. AngII and Ang (1–7) promote neuroprotection through activation of AT2R and MASR, respectively. However, AngII through activation of AT1R leads to the development of oxidative stress, inflammation, and apoptosis, and progression of neurodegeneration.

It has been shown that several brain regions, particularly the nigrostriatal pathway, contain all RAS elements, which is higher than in other peripheral organs (Babalghith et al. 2022; Moubarak et al. 2021; Al‐Kuraishy et al. 2021, 2018). The brain RAS plays a distinct role in normal physiological and pathological conditions (Figure 3) (Wright and Harding, 2019). Angiotensinogen, the precursor molecule for AngI, AngII, and AngIII, and the enzymes renin, ACE, and APM are synthesized within the brain. In addition, AT1R, AT2R, and AT4R are expressed in the brain (Pan et al. 2023). ATRs are found in several brain regions, such as the hypothalamic paraventricular and supraoptic nuclei, the lamina terminalis, the lateral parabrachial nucleus, the ventrolateral medulla, and the nucleus of the solitary tract (NTS), which are known to have roles in the regulation of the cardiovascular system and/or body fluid and electrolyte balance (Al‐Kuraishy et al. 2024; Al‐Kuraishy, Al‐Gareeb, et al. 2020). Notably, the angiotensinergic neural pathway utilizes AngII and/or AngIII as a neurotransmitter or neuromodulator in many brain regions. Angiotensinogen is synthesized predominantly in astrocytes, but the processes by which AngII is generated or incorporated into neurons for utilization as a neurotransmitter are unknown. Centrally administered AT1R antagonists or angiotensinogen antisense oligonucleotides inhibit sympathetic activity and reduce arterial blood pressure in certain physiological or pathophysiological conditions, as well as disrupt water drinking and sodium appetite, vasopressin secretion, sodium excretion, renin release, and thermoregulation (Tong et al. 2024). The AT4R is identical to insulin‐regulated aminopeptidase (IRAP) and plays a role in memory mechanisms (Pan et al. 2023; Al‐Kuraishy et al. 2024; Tong et al. 2024). Therefore, angiotensinergic neural pathways and angiotensin peptides are essential in neural and homeostatic functions, particularly related to cardiovascular function, osmoregulation, and thermoregulation. Interestingly, angiotensinogen, prorenin, and renin are unable to cross the blood‐brain barrier (BBB) (Haron et al. 2021).

Nevertheless, prorenin has been detected in the brain, although its origin has not been verified. The prorenin receptor (PRR) activation induces angiotensinogen cleavage and RAS activation (Worker et al. 2020). PRR forms the majority of AngII in the CNS and regulates blood pressure and cardiovascular function (Worker et al. 2020). It is worth mentioning that astrocytes are responsible for producing 90% of brain angiotensinogen. However, neurons and glial cells create a smaller amount of brain angiotensinogen (Gouveia et al. 2022). The angiotensinogen found in the CSF and secreted by cultures of glia and neurons is similar to the two major molecular sizes found in plasma. However, glia and neurons secrete distinct forms of angiotensinogen. The expression of different forms is under hormonal regulation (Loera‐Valencia et al. 2021; Vadhan and Speth, 2021). If these structural forms are shown to affect function, then the resulting ramifications may extend to pathological conditions, such as hypertension. Primary cell cultures of astrocytes secrete angiotensinogen constitutively and in a region‐specific manner related to the size of the subpopulation of secretory cells. Neuron cultures secrete angiotensinogen at about 25% the rate of hypothalamic astrocytes (Loera‐Valencia et al. 2021; Vadhan and Speth, 2021).

Moreover, AT1R and AT2R are highly expressed in glial cells, along with prorenin (Cosarderelioglu et al. 2020), suggesting that the RAS is involved in oxidative stress and inflammation in the brain. AT1R and AT2R, able to form heterodimers (AT_1/2_Hets), are present in the CNS and involved in the pathogenesis of PD (Rivas‐Santisteban et al. 2020). AT_1/2_Hets are innovative, efficient units with specific signaling properties. The co‐activation of the heteromer decreases the signaling output of Ang. AT_1/2_Hets, which are expressed in both striatal neurons and microglia, create the possibility that candesartan, the antagonist of AT1R, increases the effect of AT2R agonists. In addition, the level of striatal expression increased in the unilateral 6‐OH‐dopamine‐lesioned rat PD model. It was markedly higher in PD‐like animals that did not become dyskinetic upon chronic levodopa administration compared with expression in those that became dyskinetic (Rivas‐Santisteban et al. 2020; Martínez‐Pinilla et al. 2014; Rivas‐Santisteban et al. 2021, 2024; Martínez‐Pinilla et al. 2015).

Furthermore, AT_4_R is critical for dopamine and acetylcholine release and mediates learning and memory consolidation (Cosarderelioglu et al. 2020, 2023). Astrocytes and cerebral vascular endothelium also contain ACE, the primary enzyme in RAS (Gouveia et al. 2022). Brain ACE is predominantly found in regions that do not have a BBB; however, it is also present in neurons and glial cells (Marquié et al. 2020; Jackson et al. 2018). However, brain areas primarily involved in blood pressure control predominantly expressed ACE2 (Alenina and Bader, 2019). In addition, the collecting domain of ACE2 regulates the levels of some amino acids in the blood, particularly tryptophan. Therefore, it is no surprise that animals with genetic alterations in the expression of ACE2 develop diverse phenotypes ranging from hypertension and metabolic and behavioral dysfunctions, to impairments in serotonin synthesis and neurogenesis (Alenina and Bader, 2019).

Of interest, aging‐associated dysregulation of RAS may lead to adverse clinical outcomes in many neurodegenerative diseases such as Alzheimer's disease (AD) and Parkinson's disease (PD) due to induction of excessive oxidative stress, neuroinflammation, endothelial dysfunction, microglial polarization, and alterations in neurotransmitter release (Alenina and Bader, 2019; Abiodun and Ola, 2020; Bild et al. 2022; Al‐Kuraishy, Jabir, et al. 2023). Furthermore, excessive activation of the brain's RAS is linked with the development of cognitive impairment in neurodegenerative diseases, mainly in AD (Bild et al. 2022). Similarly, exaggerated RAS in the brain is implicated in other neurodegenerative diseases and neurological disorders such as multiple sclerosis (MS), amyotrophic lateral sclerosis, Huntington's disease, stroke, and traumatic brain injuries (Bild et al. 2022; Barthold et al. 2020; Szczepanska‐Sadowska et al. 2022). Consistently, targeting RAS, mainly the classical pathway, by ACE inhibitors (ACEIs) and angiotensin receptor blockers (ARBs), can mitigate cognitive impairment in AD by inhibiting oxidative stress and neuroinflammation (Barthold et al. 2020). Moreover, the enhanced activity of RAS is associated with the development and progression of PD (Sunanda et al. 2021; Jo et al. 2022). However, the association between the Ang‐A/ACE2/Alamandine/MrgD axis and the pathogenesis of PD is not fully elucidated. Hence, this review aims to investigate the potential significance of the Ang‐A/ACE2/Alamandine/MrgD axis in PD etiology. Therefore, this review aims to discuss the potential role of nonclassical pathways of RAS in the pathogenesis of PD and how targeting of this pathway is involved as a therapeutic strategy in the management of PD.

## 3 Pathogenesis of PD: An Overview

PD is a complex neurological disease characterized by the progressive loss of dopaminergic neurons (DNs) in the substantia nigra (SN). PD‐associated neurodegeneration is due to the buildup of abnormal protein aggregates known as Lewy bodies (Batiha et al. 2023). The aggregation of a specific presynaptic protein called alpha‐synuclein (α‐Syn) primarily makes Lewy bodies (Al‐Kuraishy et al. 2023). Research reports indicated that the loss of more than 70% of DNs in the SN leads to the development of PD symptoms (Al‐Kuraishy, Al‐Gareeb, Kaushik, et al. 2023). PD is distinguished by motor symptoms such as resting tremors, stiffness, postural instability, and bradykinesia (Al‐Kuraishy, Al‐Gareeb, Elewa, et al. 2023). In addition, PD has numerous non‐motor symptoms such as memory loss, cognitive deterioration, sleep difficulties, and depression that may occur several years before the onset of motor symptoms (Alrouji et al. 2023b).

The aging process is the primary factor that predisposes individuals to the development of PD (Alrouji et al. 2023b). PD approximately affects 1%–3% of the general population aged 65 years or older. The prevalence of PD is higher in men than women due to the neuroprotective effect of estrogen (Alrouji et al. 2024; Balestrino and Schapira, 2020).

**FIGURE 4:**
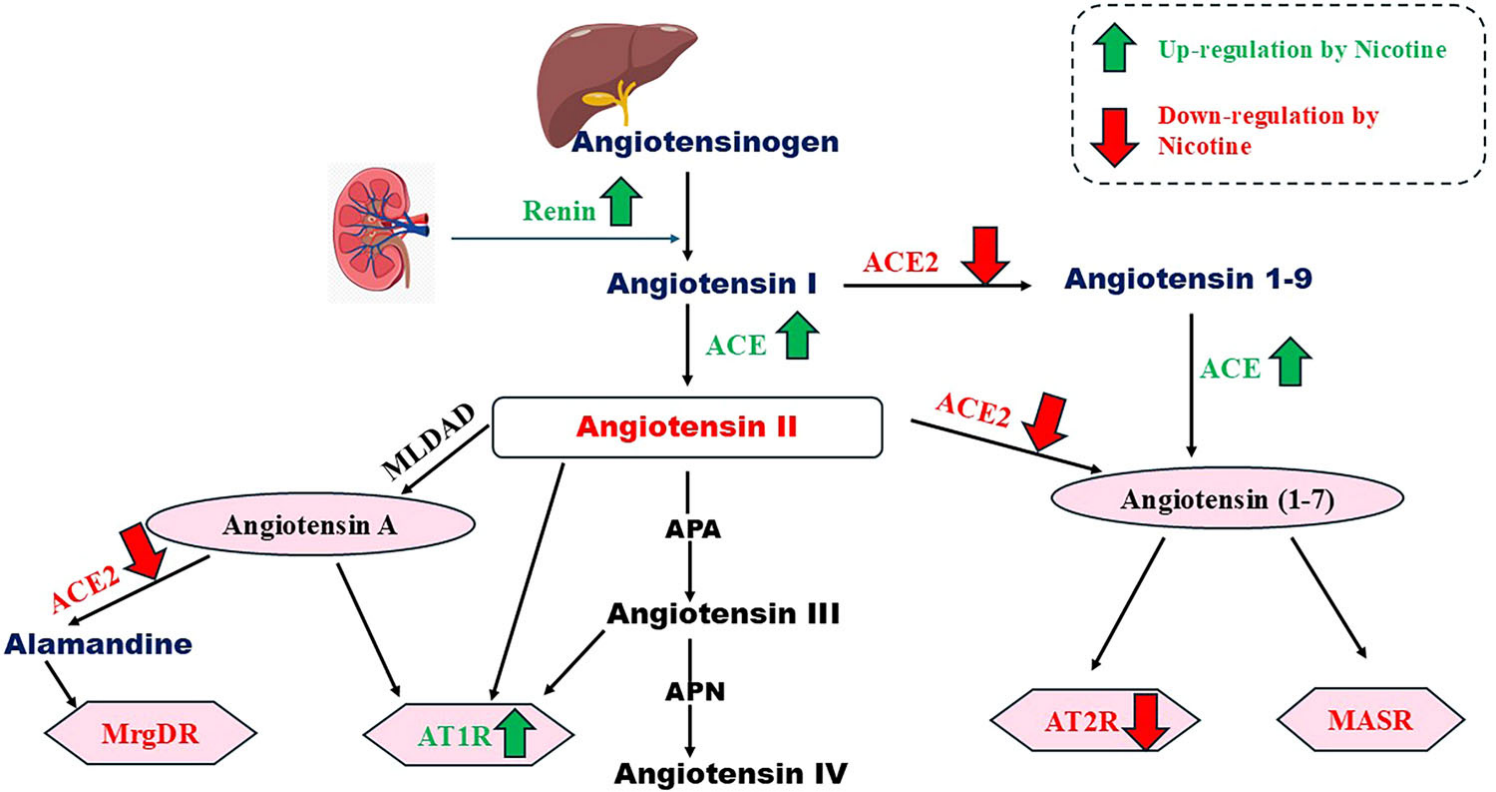
Pathophysiology of PD: Interaction of environmental factors, such as neurotoxins, with genetic factors, such as mutations of *PARKIN, DJ‐1*, *and UCH‐L1* genes, triggers the accumulation of mutant α‐Syn and the formation of Lewy bodies. These neuropathological alterations inhibit autophagy and lysosome clearance of α‐Syn with subsequent accumulation of α‐Syn and induction of neurodegeneration. Loss of 70%–90% of the DNs in the SN initiates the development of motor symptoms in PD. However, non‐motor symptoms of PD develop in the early stage of PD neuropathology.

PD is categorized into two distinct subtypes: idiopathic (sporadic) PD, which accounts for 90% of cases, and familial PD, which accounts for only 10% of cases (Al‐Kuraishy, Alexiou, et al. 2023). Researchers have linked the incidence of familial PD to the overproduction and accumulation of mutated α‐Syn (Zesiewicz, 2019). Genetic changes, in conjunction with several environmental variables, can influence the pathophysiology of PD (Alrouji et al. 2023a). Scientists have proposed that the development of PD involves three distinct temporal phases: triggers (such as environmental contaminants), facilitators (such as peripheral inflammation), and aggravators (such as autophagy failure) (Balestrino and Schapira, 2020; Alrouji, Al‐Mahammadawy, et al. 2023; Armstrong and Okun, 2020; Al‐Kuraishy, Abdulhadi, et al. 2020). Environmental factors, through interaction with genetic genes, affect the production and the elimination of α‐Syn (Balestrino and Schapira, 2020; Alrouji, Al‐Mahammadawy, et al. 2023; Armstrong and Okun, 2020; Al‐Kuraishy, Abdulhadi, et al. 2020). These neuropathological alterations trigger the development of mitochondrial dysfunction, oxidative stress, inflammation, and endoplasmic reticulum (ER) stress in the DNs of the SN (Isroilovich et al. 2022). Hence, the development of PD is complex and related to the numerous genetic and environmental factors (Figure 4).

## 4 Role of RAS in the Pathogenesis of PD

### 4.1 General Aspects

It has been reported that Allen et al. first identified the association between PD and the brain RAS in 1992 (Allen et al. 1992). They found that RAS is overexpressed in neighboring DNs of the SN cell bodies. Furthermore, they detected angiotensin receptors in the presynaptic region, suggesting that ARBs and angiotensin‐converting enzyme inhibitors (ACEIs) could potentially influence dopamine production and release (Allen et al. 1992). Furthermore, experimental and clinical research provides evidence that supports the correlation link between brain RAS and PD (Armando et al. 2004; J. Yang et al. 2022). The importance of nigrostriatal RAS has only begun to be unraveled due to a complex feedback cycle between RAS and DNs of the SN that increases the vulnerability of DNs. Consistently, inhibition of brain RAS by ACEIs and ARBs attenuates the progression of PD neuropathology by reducing oxidative stress and neuroinflammation (Al‐Qahtani et al. 2024; Sunanda et al. 2021). RAS inhibitors, particularly AT1R antagonists, attenuate DN degeneration in the MPTP‐induced model of PD (Sekar et al. 2018). Alterations in the RAS pathway have also been observed over the years in the postmortem brain of PD patients. It has been shown that a 90% decrease in AT1R binding in the SN and 70% in the caudate and putamen nuclei was detected in human postmortem PD brain tissues (Ge and Barnes, 1996). Another study established reduced AT1R immunoreactivity in PD patients compared to healthy controls, and this decrease was attributed to the loss of DNs and diminution in AT1R binding in the neurons (Zawada et al. 2015). It has been revealed that an exaggerated brain RAS is associated with the disruption of both motor and non‐motor symptoms in PD patients (J. Yang et al. 2022). A longitudinal study observed that ARBs, but not ACEIs, enhance cognitive performance in PD patients with hypertension (J. Yang et al. 2022). This result suggests that ARBs are superior to other antihypertensive drugs in their ability to ameliorate cognitive impairments in PD. In a retrospective cohort study, hypertensive patients treated with ARBs for 8.4 years illustrated that 1.1% of patients developed PD compared to 2.2% among those who did not receive ARBs (Labandeira et al. 2022). ARBs reduced PD risk more than ACEIs because they specifically block AT1R and boost the neuroprotective ACE2/Ang1–7/MASR axis (Labandeira et al. 2022). Additionally, ARBs augment the metabolism of AngII into AngIV, which has a neuroprotective effect (Gironacci et al. 2018). However, AngIII and AngIV, derived from APA and APM from Ang‐A, lead to endothelial dysfunction and inflammation (Hrenak et al. 2016).

Furthermore, research links peripheral blood mononuclear cell (PBMC) infiltration into the CNS with the development of neuro‐inflammation and the progression of PD (Kyrkanides et al. 2008). Increasing peripheral infiltration of leukocytes into the brain may be protective for the resolution of neuro‐inflammation in different neurodegenerative disorders (Schwartz and Baruch 2014). Higher levels of IL‐1β in the brains of mice with subclinical neurodegeneration promote leukocyte entry into the brain across the deranged BBB (Shaftel et al. 2007). The migration of leukocytes from the blood vessels into the brain causes the degeneration of DNs in the SN. Consequently, an increased presence of leukocytes that contain MLDAD in the SN facilitates the transformation of AngII into Ang‐A, resulting in the deterioration of DNs and the onset of PD (Rodriguez‐Pallares et al. 2004).

### 4.2 Specific Aspects

#### 4.2.1 Role of Ang‐A in PD

In 2007, Ang‐A was first identified by Jankowski et al. (2007) as a vasoconstrictor octapeptide in human plasma. Ang‐A differs from AngII by a single amino acid, alanine, instead of asparagine, so‐called Ang alanine (Jankowski et al. 2007). Patients with renal failure exhibit an increase in Ang‐A/AngII, despite Ang‐A being normal and present in the plasma at a 20% lower level than AngII (Jankowski et al. 2007). The MLDAD enzyme primarily derives Ang‐A from AngII, though ACE2 further metabolizes Ang‐A to alamandine (Oakes et al. 2018). Ang‐A is synthesized by the enzymatic decarboxylation of Asp^1^ of the AngII sequence. Ang‐A is a potent vasoconstrictor similar to AngII. Ang‐A activates both AT1R and AT2R, though it mainly stimulates AT1R (R. Yang et al. 2011). However, the vasoconstrictor effect of Ang‐A, which activates AT1R, necessitates a higher concentration than that of Ang‐II (R. Yang et al. 2011; Habiyakare et al. 2014). Furthermore, AT1R antagonist losartan attenuates Ang‐A‐induced vasoconstriction (R. Yang et al. 2011). An experimental study revealed that Ang‐A administration by intravenous route can induce a dose‐dependent increase in blood pressure due to vasoconstriction (Coutinho et al. 2014). In theory, ARBs raised AngII because they blocked negative feedback loops produced by Ang‐A. That may have a greater effect on the neuropathology of PD by activating AT1R in the DNs of the SN (J. Yang et al. 2022).

It has been reported that ARBs lessen the effect of Ang‐A and AngII by inhibiting AT1R in the DNs of the SN (Labandeira et al. 2022). However, while endopeptidase and chymase convert AngI to AngII independently of ACE, ACEIs that inhibit AngII also reduce Ang‐A (Garrido‐Gil et al. 2012; Sumien et al. 2021; Tonneijck et al. 2014). According to reports, endopeptidase and chymase show high levels of expression in the DNs of the SN (Zhang et al. 2021; Labandeira‐Garcia et al. 2012). This study's findings may explain why ARBs have a greater ability to protect the brain in PD compared to ACEIs. Based on these findings, it seems that Ang‐A is a key factor in the development of PD, and the use of ARBs and ACEIs to control the functional activity of Ang‐A could help to treat PD.

#### 4.2.2 Role of Alamandine in PD

Alamandine is a vasoactive heptapeptide with similar effects and structure to Ang1–7 but differs by only one amino acid, aspartate, replaced with alanine. Alamandine is formed from Ang‐A by the effect of ACE2 and directly from Ang1–7 (Villela et al. 2014). Alamandine acts by activating the MrgD receptor, which Ang1–7 also activates (Villela et al. 2014). Two independent researchers discovered the MrgD receptor in 2001, initially identifying receptors related to MASR as sensory‐specific G protein‐coupled receptors (Dong et al. 2001; Lembo et al. 2002). AngIII and AngIV, derived from Ang‐A, also activate the MrgD receptor (Gembardt et al. 2008). Different tissues, including the lung, thymus, cerebellum, prostate, skeletal muscles, and adipose tissue, highly express the MrgD receptor (Avula et al. 2013). MrgD activates the expression of endothelial nitric oxide synthase (eNOS) (Habiyakare et al. 2014). As a result, the alamandine/MrgD axis represents the protective axis of RAS (Schleifenbaum, 2019). Studies have demonstrated that alamandine, through increasing the release of acetylcholine, attenuates AngII‐induced vasoconstriction more effectively than Ang1–7 (Lautner et al. 2013). A preclinical study also confirmed that alamandine causes vasodilation in MASR‐knockout mice (Lautner et al. 2013), which shows that alamandine works differently from that of the Ang1–7/MASR axis. Consistently, MASR antagonists abolish the effect of Ang1–7 but not alamandine (Villela et al. 2014).

Moreover, the expression and functional role of ACE2 and the alamandine/MrgD axis are reduced in the SN and predispose to the degeneration of DNs during aging (Valenzuela et al. 2021). In addition to MrgD, alamandine promotes neuronal mitochondrial function via activation of MrgE in the mitochondrial membrane and the expression of neuronal NO (Valenzuela et al. 2021). Worth mentioning, the expression of ACE2 is highly reduced in PD (Angelopoulou et al. 2023), leading to a significant reduction of Ang1–7 and the formation of alamandine. It has been illustrated that the ACE2/Ang1–7/alamandine axis is highly dysregulated, leading to the degeneration of DNs in the SN in PD (lmeida‐Santos et al. 2017). Increased RAS activity leading to an increase in AngII levels may increase the risk of developing PD and other neurodegenerative diseases. However, the alteration in the balance among angiotensin peptides, resulting in increasing Ang (1–7) or alamandine, may represent an effective neuroprotective strategy in population groups at high risk or as an adjunctive treatment to reduce the progression of PD (lmeida‐Santos et al. 2017). The neuroprotective effect of Ang1–7 and alamandine against PD neuropathology is related to the inhibition of the aggregation of α‐Syn (Gao et al. 2022). A recent experimental study illustrated that administering Ang1–7 into the SN for 4 weeks in rats mitigates PD motor symptoms (Gao et al. 2022). Besides, an in vitro study revealed that Ang1–7 reduced α‐Syn accumulation by enhancing autophagy in the DNs subjected to neurotoxins (Gao et al. 2022).

Furthermore, preclinical research discovered that Ang1–7 activates MASR and inhibits NADPH oxidase, leading to anti‐inflammatory and antioxidant effects with consequent preservation of DNs in the SN (Rabie et al. 2018). Therefore, the augmentation of Ang1–7 and alamandine serves as a preventive measure against the deterioration of DNs in the SN. Moreover, ACEIs and ARBs promote the production of ACE2, resulting in the formation of Ang1–7 and alamandine. Therefore, the ability of ACEIs and ARBs to protect against PD is associated with their ability to block the traditional RAS pathway and stimulate the ACE2/Ang1–7/alamandine/MrgD axis (Kuba et al. 2010; Gong et al. 2022).

Furthermore, the neuroprotective effect of alamandine against the progression of PD is through suppression of oxidative stress. Oxidative stress causes DNs to deteriorate in the SN by stimulating an inflammatory response and activating inflammatory signaling pathways (Blesa et al. 2015). Furthermore, alamandine counteracts the AngII‐induced vascular fibrosis by stimulating MrgD and suppressing p38 mitogen‐activated protein kinase (p38MAPK) activity (C. Yang et al. 2020). Various investigations have shown a correlation between elevated brain RAS and p38MAPK and the onset of PD (Parga et al. 2021; Obergasteiger et al. 2018). Alamandine has a neuroprotective effect similar to Ang (1–7) through MrgDR activation, distinct from MasR. It reinforces MasR‐MrgDR interaction and induces anti‐inflammatory and antiproliferative responses in human macrophages (Rukavina Mikusic et al. 2024). Furthermore, alamandine improves the expression of brain‐derived neurotrophic factor (BDNF), which has a neuroprotective effect against the development and progression of PD (Scalzo et al. 2010; Becari et al. 2023). Thus, alamandine may be effective in treating PD by mitigating inflammatory and oxidative stress and BDNF expression.

Taken together, Ang‐A and Ang1–7/alamandine show contrasting effects on PD neuropathology (Table 2). Therefore, increasing Ang1–7/alamandine and reducing Ang‐A could be a new and effective treatment of PD. The present review has several limitations, including that most of the findings are derived from preclinical studies that have not been entirely translated into clinical studies. That represents the chief gap, along with a consideration of potential clinical applications. Despite these limitations, the present review gave a clue for targeting the brain RAS in managing PD. For example, activation of AngII/AT2R, ACE2/Ang1–7, and Ang1–7/alamandine, or inhibition of AngII/AT1R and related enzymatic pathways, could effectively reduce PD's development and progression. Therefore, further preclinical trials, clinical trials, and clinical studies are recommended in this regard.

**TABLE 2:** Role of the nonclassical pathway of RAS in PD.

Type of the study	Findings	Mechanisms	Ref.
Preclinical Preclinical Preclinical Preclinical Preclinical Preclinical Preclinical	ARBs attenuate the effect of Ang‐A and AngII. ACE2 and the alamandine/MrgD axis are reduced in the SN and predispose to the degeneration of DNs during aging. Alamandine promotes neuronal mitochondrial function. The expression of ACE2 is highly reduced in PD. Increasing Ang (1–7) or alamandine reduces the progression of PD. Ang1–7 and alamandine mitigate PD neuropathology. Ang1–7 preserves DNs in the SN.	Inhibition of AT1R in the DNs of the SN. Augmentation of inflammation and oxidative stress. Activation of MrgE in the mitochondrial membrane and the expression of neuronal NO. Reduction of Ang1–7 and the formation of alamandine. Ang (1–7) or alamandine has anti‐inflammatory and antioxidant effects. Inhibition of the aggregation of α‐Syn. Ang1–7 inhibits NADPH oxidase, leading to anti‐inflammatory and antioxidant effects.	Labandeira et al. (2022). Valenzuela et al. (2021). Valenzuela et al. (2021). Angelopoulou et al. (2023). lmeida‐Santos et al. (2017). Gao et al. (2022). Rabie et al. (2018).

## 5 Conclusions and Future Perspectives

PD is a complex neurological disease characterized by the progressive loss of DNs in the SN. PD‐associated neurodegeneration is due to progressive accumulation of α‐Syn in the DNs of SN. Overexpression and overactivity of the classical pathway of RAS in the nigrostriatal pathway are linked with the progression of PD by inducing inflammation and oxidative stress. Nevertheless, the nonclassical pathway of RAS, including AngII/AT2R, ACE2/Ang1–7, and Ang1–7/alamandine, has protective effects against the development and progression of PD by counteracting the deleterious effects of the classical pathway of RAS. Therefore, therapeutic strategies focusing on enhancing alamandine signaling and suppressing Ang‐A activity represent promising approaches for PD management. Future research should prioritize the clinical translation of these findings. However, the protective role of the nonclassical pathway of RAS in PD came from preclinical studies that have not entirely translated into clinical studies. Importantly, future studies examining the use of ACE2/Ang1–7 and Ang1–7/alamandine activators may open a new avenue in the management of PD. Thus, further clinical trials and prospective studies are recommended in this regard.

## Author Contributions

**Ghadah H. Alshehri**: writing – review and editing, validation. **Hayder M. Al‐kuraishy**: conceptualization, data curation, writing – original draft. **Ali K. Albuhadily**: conceptualization, data curation, writing – original draft. **Ali I. Al‐Gareeb**: conceptualization, data curation, writing – original draft. **Amany S. Aboutaleb**: visualization, writing – review and editing. **Athanasios Alexiou**: validation, writing – review and editing. **Marios Papadakis**: writing – review and editing, validation. **Gaber El‐Saber Batiha**: project administration, supervision.

## Ethics Statement

The authors have nothing to report.

## Consent

The authors have nothing to report.

## Conflicts of Interest

The authors declare no conflicts of interest.

## Peer Review

The peer review history for this article is available at https://publons.com/publon/10.1002/brb3.70721.

## Data Availability

The authors have nothing to report.
